# c-Myb Regulates Cell Cycle-Dependent Expression of Erbin: An Implication for a Novel Function of Erbin

**DOI:** 10.1371/journal.pone.0042903

**Published:** 2012-08-07

**Authors:** Dan Liu, Ming Shi, Hao Zhang, Lu Qian, Ming Yu, Meiru Hu, Ruihong Zhang, Tianyou Wang, Caili Han, Huijun Duan, Ning Guo

**Affiliations:** 1 Department of Pathology, Hebei Medical University, Shijiazhuang, People's Republic of China; 2 Department of Pathophysiology, Institute of Basic Medical Sciences, Beijing, People's Republic of China; 3 Capital Institute of Pediatrics, Beijing, People's Republic of China; Institut de Génétique et Développement de Rennes, France

## Abstract

In the present study, we demonstrated the cell cycle periodicity of Erbin expression with the maximal expression of Erbin in G2/M phase. A significant increase in Erbin promoter activity was observed in G2/M phase-synchronized cells. Sequence analysis revealed a c-Myb site in the core promoter region of *Erbin*. Mutagenesis of c-Myb consensus sequences abrogated the increased *Erbin* promoter activity in G2/M phase. ChIP and oligonucleotide pull-down assays validated that the recruitment of c-Myb to the consensus sequences was specific. The interaction of c-Myb with c-Myb site in the *Erbin* promoter was significantly enhanced in G2/M phase. Ectopic overexpression of c-Myb led to the up-regulation of *Erbin* promoter activity and c-Myb silencing by small interfering RNA significantly decreased Erbin protein level. Transfection of c-Myb rescued Erbin expression that was impaired by c-Myb knockdown. It proves that c-Myb and the c-Myb response element mediate the cell cycle-dependent expression of Erbin. Inactivation of Erbin causes an acceleration of the G1/S transition, the formation of multipolar spindles and abnormal chromosome congression. These results unravel a critical role of c-Myb in promoting *Erbin* transcription in G2/M phase and also predict an unappreciated function of Erbin in cell cycle progression.

## Introduction

Erbin belongs to the LAP [LRR (leucine-rich repeats) and PDZ (PSD-95/Discslarge/ZO-1)] protein superfamily [1], [2]. The structure of Erbin is characterized by two identifiable domains: 16 LRR motifs (residues 23–391) and a single PDZ domain (residues 1, 280-1, 368). A LRR-like domain (residues 392–429) and an intermediary region containing proline rich stretches are located between the LRR and PDZ domains. LAP proteins are generally localized at the basolateral membrane or associated with lateral junctions in polarized epithelial cells of worms, flies and humans, indicating a critical role of this protein family in generating membrane asymmetry and assembling the individual cells into three dimensional tissues of animals [3]–[6].

Erbin was originally described as a Her2-binding partner. It was known that Erbin was constitutively associated with Her2 receptor and directly bound to the C terminus of Her2 in living cells, guiding the basolateral localization of Her2 [1]. Discoveries of various Erbin binding partners by later investigations indicate the functional roles of Erbin in determining cell polarity and cell adhesion [7]–[10], since the binding partners of Erbin are mainly the proteins that are the components of adherens junctions, such as p120 catenin family proteins p0071 and δ-catenin, plakophilin-related armadillo-repeat protein-interacting protein, armadillo repeat gene deleted in velocardiofacial syndrome, and the proteins involved in cell attachment to substrates, such as β4-integrin and bullous pemphigoid antigen 1 [5], [8]–[11]. Several studies reveal that Erbin also acts as a signaling molecule, exerting negative regulatory functions in different signaling pathways, including mitogen-activated protein kinase (MAPK), nuclear factor-κB (NF-κB) and transforming growth factor β (TGF-β) pathways [12]–[18]. Our recent findings demonstrate that Erbin exerts dual functions in ERK signaling pathway in cardiomyocytes, either as a negative regulator to suppress EGF-induced ERK activation or as a positive regulator to enhance catecholamine-stimulated ERK activation [19]. However, the functions of Erbin have not been extensively investigated so far.

Like other members of the LAP family, Erbin is predominantly localized at the basolateral membrane or lateral junctions in polarized epithelial cells. However, we noticed that Erbin was exceptionally aggregated in the nuclei of mitotic cells with remarkably increased abundance at G2/M stage. As a matter of fact, the nuclear localization of Erbin in human keratinocytes could be visualized but ignored in an earlier study [20]. The data suggest an unappreciated function of Erbin in cell cycle progression. So far, the potential relevance of the Erbin expression to mitosis has been unknown and the regulatory mechanisms of the Erbin expression unexplored.

In the present study, we demonstrate that c-Myb is a strong transactivator engaged in the cell cycle-dependent expression of Erbin. Our data implicate that Erbin may be involved in the regulation of cell cycle transition.

## Materials and Methods

### Cell culture and synchronization

Human breast cancer cell lines SKBR3 and MCF-7, human cervical carcinoma cell line HeLa, and human kidney cell line 293T are obtained from American Type Culture Collection (ATCC). Human normal liver cell lines LO2 and HL-7702 were purchased from the Shanghai Institute of Cell Biology of the Chinese Academy of Science. The cells were maintained in Dulbecco's modified Eagle's medium (Invitrogen) supplemented with 10% fetal bovine serum (HyClone). For synchronization, cells grown in 24-well plates with an initial cell density of 1×10^5^ cells/well were blocked for 16 h with 2 mM thymidine (Sigma), released for 8 h by washing out the thymidine with phosphate-buffered saline (PBS), and then blocked again with 2 mM thymidine for 16 h to synchronize cells at G1/S boundary. This time point was designated 0 h. To obtain S phase-arrested cells, cells were synchronized by a double-thymidine block and harvested at 5 or 6 h after release. For G2/M phase block, cells were treated with 0.4–0.8 µg/ml nocodazole for 16 h. Cells were also synchronized by 400 µM mimosine for late G1 phase arrest. The cell cycle distribution of the population was determined by propidium iodide staining and flow cytometry (FACSCalibur system, BD Biosciences).

### Plasmid construction

The 5′-flanking region of human *Erbin* gene, spanning from −661 to +44 bp relative to transcription initiation site (GenBank accession number NT 006713.15) was amplified from the genomic DNA of HeLa cells by polymerase chain reaction (PCR) with the primer P1 containing an *Xho* I site and P2 a *Hin*d III site using Power Pfu DNA polymerase (BioTeke). The resultant DNA fragment was cloned into the *Hin*d III and *Xho* I sites at immediate upstream of a firefly luciferase gene in pGL3-Basic reporter vector designated pLuc-661. A series of deletions from the 5′-end of the *Erbin* promoter were amplified with the distinct 5′ primers P3, P4, P5, P6 and P7 (Table 1) and a common 3′ primer P2 using pLuc-661 as a template. The PCR products carrying 5′ *Xho* I and 3′ *Hin*d III sites were also cloned into the plasmid pGL3-Basic to generate pLuc-483, pLuc-341, pLuc-271, pLuc-232 and pLuc-176.

**Table 1:** Primers used.

Application	Primer	Sequence (5′→3′)
pLuc-661	P1	a ccgctcgagatcttcgtcttattagag
	P2	b cccaagcttcaagagtagtgacagtctcc
pLuc-483	P3	a ccgctcgagcgagtagctgggactac
pLuc-341	P4	a ccgctcgagggattacaggtgtgagc
pLuc-271	P5	a ccgctcgagtcatggattgctttgt
pLuc-232	P6	a ccgctcgagaatttgaaatccagtag
pLuc-176	P7	a ccgctcgagattgtgcttgtatagtg
Point mutation of AP-1	P8	gataaacacttctcctgccactataca
	P9	gcaggagaagtgtttatctgtgacatatga
Point mutation of mc-Myb	P10	aacaaatcattccaatctatcacaat
	P11	agattggaatgatttgttctgaaaaaccgttgtaatatg
pHA-c-Myb	P12	a ccgctcgagatggcccgaagaccccggcacagca
	P13	c cggggtacctcacatgaccagcgtccgggctga
RT-PCR of Erbin	P14	atattgttaaccatgatgatgt
	P15	agcgattagttctaattgagaaaa
RT-PCR of β-actin	P16	gtggggcgccccaggcacca
	P17	cttccttaatgtcacgcacgatttc
ChIP assay of Erbin	P18	tgtatagtggcaggagaagtgt
	P19	caaccgcacaaacaaacttcgt

a *Xho* I,

b *Hin*d III, and

c *Kpn* I sites are underlined.

Site-directed mutagenesis in the potential AP-1 and c-Myb binding sites at the positions −131/−141 and −86/−103 of the *Erbin* promoter were generated by overlapping PCR with pLuc-483 as a template using the primers P2, P3, P8–P11 and TaKaRa mutanBEST kit. The resulting constructs were designated pLuc-mAP-1 and pLuc-mc-Myb.

The open reading frame sequence of human *c-Myb* (GenBank accession number NM 001130172.1) was obtained by reverse transcription PCR (RT-PCR) with HeLa mRNA as a template using the primers P12 and P13 and reverse transcription kit (BioTeke). The PCR product was cloned into the plasmid pXJHA designated pHA-c-Myb.

The sequences, 5′ - aaggacagcagacacagaacc - 3′ and 5′ - cctcttagaatttgcagaaac - 3′, in c-Myb mRNA were selected through the siRNA Target Finder (http://www.ambion.com/techlib/misc/siRNA_finder.html, Ambion) as siRNA target sites and expressed by using the GeneSuppressor system (Imgenex). The synthetic double-strand oligonucleotides (c-Mybsh1, sense: 5′ – tcgaaaggacagcagacacagaaccggaattcgggttctgtgtctgctgtccttttttt – 3′ and antisense: 5′ – ctagaaaaaaaggacagcagacacagaacccgaattccggttctgtgtctgctgtcctt – 3′; c-Mybsh2, sense: 5′ – tcgacctcttagaatttgcagaaacggaattcggtttctgcaaattctaagaggttttt – 3′ and antisense: 5′ – ctagaaaaacctcttagaatttgcagaaaccgaattccgtttctgcaaattctaagagg – 3′; GFP shRNA used as a control, sense: 5′ –tcgagagagaccacatggtccttctggaattcgagaaggaccatgtggtctctcttttt – 3′ and antisense: 5′ –ctagaaaaagagagaccacatggtccttctcgaattccagaaggaccatgtggtctctc – 3′) were inserted into pSuppressorNeo plasmid (Imgenex), respectively, according to the manufacturer's recommendation. All constructs were verified by sequence analysis.

### Transfection and luciferase assays

Transfection was carried out using Lipofectamine 2000 (Invitrogen) according to the manufacturer's protocol. For reporter assay, cells grown in 24-well plates (1.2×10^5^ per well) were transiently transfected with 0.8 µg of the luciferase reporter construct and 0.008 µg of the plasmid pRL-TK (Promega) as an internal control. For evaluating the effect of c-Myb on the *Erbin* promoter activities, HeLa cells were co-transfected with 0.25 µg reporter plasmid, 0.55 µg effector plasmid p-HA-c-Myb and 0.008 µg of pRL-TK. At 5 h after transfection, the media were replaced and cells incubated for an additional 48 h. Transfectants were lysed and assayed for firefly and renilla luciferase activities with a dual luciferase assay kit (Promega) according to the manufacturer's instructions. Luciferase activity was calculated by dividing firefly luciferase activity by renilla luciferase activity in each sample. All transfections were carried out in triplicate and repeated at least three times.

### Immunofluorescence and confocal microscopy

The cells were grown on 35 mm dishes, washed and fixed in 4% paraformaldehyde. The fixed cells were then permeablized with methanol. After washing with PBS, the cells were blocked with 3% BSA in PBS, incubated with the mouse monoclonal antibody against Erbin [21], rinsed with PBS, and then treated with fluorescein isothiocyanate (FITC)-conjugated goat-anti-mouse antibody. After washing with PBS, the cells were treated with the solution containing 1 µg/ml of DAPI (Sigma) and observed under a laser scanning confocal microscope (RADIANCE 2100, BioRad). For determining the number of cells at different phases of mitosis, cells were fixed, stained by 5 µg/ml propidium iodide and photographed. Cells in the mitosis phase were counted and divided by the total amount of observed cells.

### Real-time RT-PCR

Total RNA was isolated from HeLa cells using the TRIzol reagents (Invitrogen) following the manufacturer's instructions and quantified by spectrophotometry. cDNA was synthesized from 5 µg of total RNA using reverse transcription kit (BioTeke) in accordance with the manufacturer's instructions and subsequently amplified by using the primers P14 and P15 specific for *Erbin* and Power DNA polymerase (BioTeke). Real-time PCR was performed using SYBR Green Supermix (TransGen Biotech) on Real-Time PCR Detection System (Eppendorf) as recommended by the manufacturer. The results were analyzed using the comparative threshold cycle method with β-actin as an internal control. The experiment was conducted in triplicate.

### Western blot

The whole cell lysates were prepared, separated by SDS-PAGE and transferred to PVDF membranes. After blocking, blots were probed with the appropriate primary antibodies overnight at 4°C. The antibodies used include anti-Erbin mouse polyclonal antibody [21], anti-c-Myb rabbit monoclonal antibody (Santa Cruz Biotechnology, INC.), anti-HA rabbit polyclonal antibody (Abcam) and anti-glyceraldehyde-3-phosphate dehydrogenase (GAPDH) rabbit monoclonal antibody (Cell Signaling Technology Inc.). The blots were then washed and incubated with horseradish peroxidase-conjugated secondary antibodies. Bands were detected by enhanced chemiluminesence (Pierce).

### Chromatin immunoprecipitation (ChIP)

The ChIP assay was performed using the SimpleChIP™ Enzymatic Chromatin IP Kit (Cell Signaling Technology Inc.) according to the protocol provided by the manufacturer. Briefly, 4×10^7^ cells, transfected with or without pHA-c-Myb, were synchronized at G1 or G2/M phase. The cells were then collected, cross-linked with 1% formaldehyde, washed once with cold PBS containing protease inhibitors and lysed. Nuclei were prepared and chromatin digested by nuclease S7 to a length of approximately 150–900 bp. HA-c-Myb/DNA or endogenous c-Myb/DNA complexes were precipitated by anti-HA antibody (Abcam) or by anti-Myb antibody (Santa Cruz). Rabbit IgG was used as a negative control. The precipitated DNA was amplified by PCR using primers P18 and P19 flanking the c-Myb binding site (−103 to −86) in the *Erbin* promoter. Final products were resolved on a 1% agarose gel.

### Preparation for nuclear extracts and oligonucleotide pull-down assays

The 5′ – biotinylated double-stranded oligonucleotides (5′ – gttctgaaaaaccgttgtaatatgtatgtt – 3′ and 5′ – aacatacattacaacggtttttcagaac – 3′) corresponding to positions −106 to −77 in the *Erbin* promoter harboring the c-Myb motif were synthesized by Invitrogen Biotechnology. The same double-stranded sequences that are not biotinylated were used as the competitors. The biotinylated oligonucleotides containing a mutated c-Myb binding site (5′ – gttctgaaaaaccatcgcaatatgtatgtt – 3′ and 5′ – aacatacatattgcgatggtttttcagaac – 3′), in which conserved nucleotides of c-Myb consensus sequence was replaced (underlined), and the biotinylated oligonucleotides (5′ – tgagtactaagaagcagactcaaagcacag – 3′ and 5′ – ctgtgctttgagtctgcttcttagtactca – 3′) corresponding to positions −633 to −604 bp of the *Erbin* promoter lacking the c-Myb binding site were also synthesized. The nuclear extracts of HeLa cells were prepared by using a Nuclear-Cytosol Extraction Kit (Applygen Technologies) according to the manufacturer's instructions. A total of 200 µg of nuclear extracts was incubated at 4°C for 4 h with each pair of the oligonucleotides previously coupled to Dynabeads M-280 (Invitrogen). The protein-DNA complexes were separated with a Dynal magnet, denatured in SDS sample buffer, and subjected to SDS-PAGE. c-Myb was detected by Western blot.

### Statistical analysis

Statistical analysis was performed using two-way analysis of variance (ANOVA) test. *P*<0.05 is considered as statistically significant.

## Results

### Erbin is expressed in a cell cycle-dependent manner

**Figure 1:**
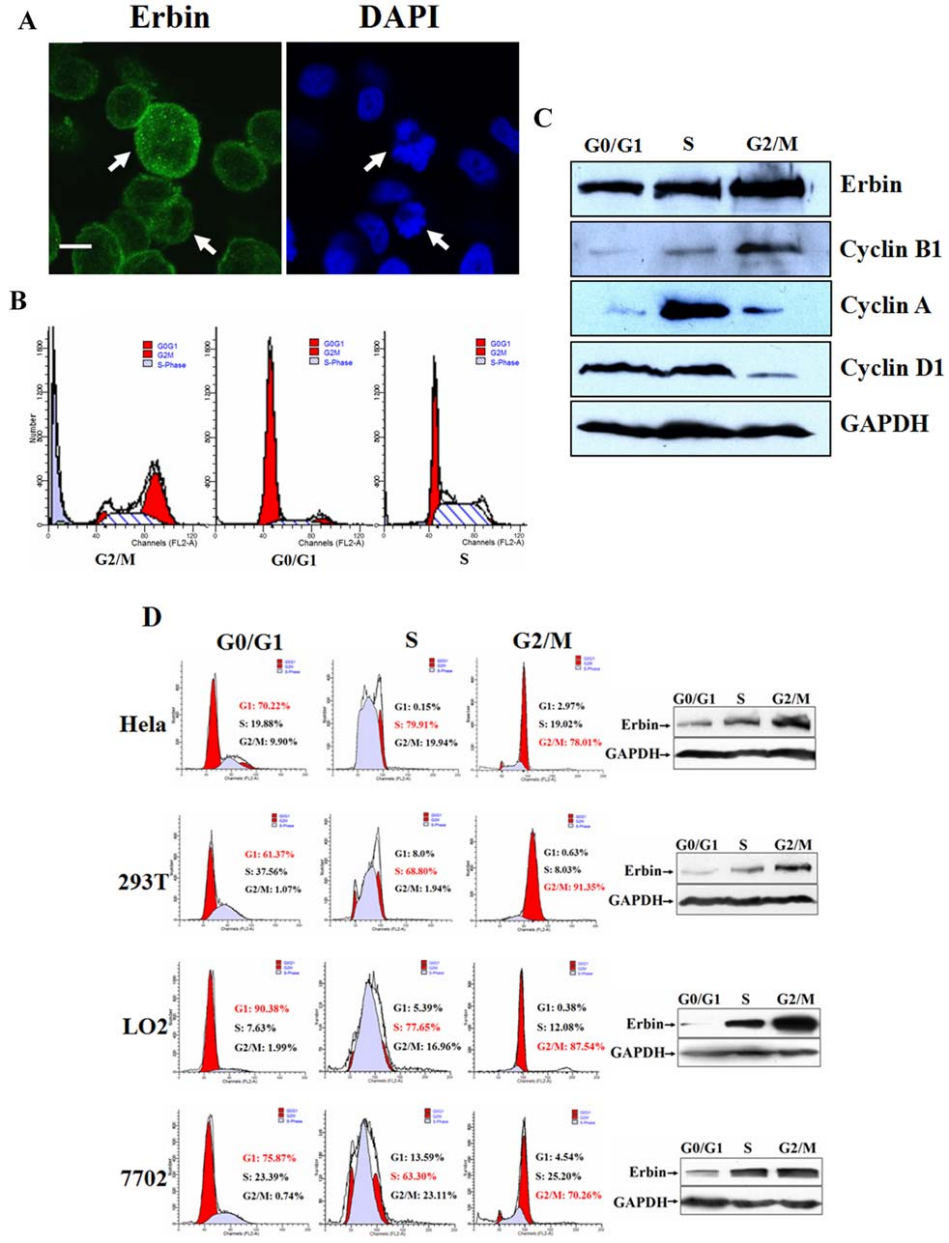
Erbin protein expression is cell cycle-dependent. A, SKBR3 cells were stained by immunofluorescent method using anti-Erbin antibody. DAPI staining was utilized to display the nuclei. The stained cells were observed under a laser scanning confocal microscope. Bar = 10 µm. B, celllular DNA content was analyzed by flow cytometry. C, MCF-7 cells were arrested at G2/M phase using nocodazole followed by their release into the cell cycle. Cells at different stages of cell cycle were lysed and whole-cell lysates subjected to SDS-PAGE and Western blot analysis with anti-Erbin antibody. Analysis of cyclin A, cyclin B1 and cyclin D1 were utilized to dissect cell cycle progression. D, HeLa, 293T, LO2 and HL-7702 cells were synchronized by a double-thymidine block or nocodazole treatment and cellular DNA content was analyzed by flow cytometric analysis and Erbin expression detected by Western blot.

A previous report showed that Erbin is redistributed from the plasma membrane into the cytosol in basal cell carcinoma [20]. Since Erbin can bind to the C terminus of Her2 receptor that is frequently overexpressed in human breast cancer cells, we therefore sought to explore the distribution of Erbin in human breast cancer cells overexpressing Her2. We stained SKBR3 cells by immunofluorescent method using a specific antibody against Erbin. DAPI staining was utilized to display the nuclei. As exhibited in Fig. 1A, Erbin was diffusely distributed near the cytoplasmic membrane in SKBR3 cells. Surprisingly, we found that the expression of Erbin was strikingly increased in the mitotic cells. This finding led us to speculate the correlation of Erbin with mitosis. We monitored the level of Erbin throughout the cell cycle by cell cycle synchronization. Using nocodazole, a microtubule inhibitor, MCF-7 cells were arrested at G2/M phase and then released into cell cycle synchronously. Cell cycle arrest was confirmed by flow cytometric analysis of cellular DNA content (Fig. 1B). Cells at different stages of mitosis were lysed and whole-cell lysates subjected to SDS-PAGE and Western blot analysis with the anti-Erbin antibody. Analysis of cyclin A, cyclin B1 and cyclin D1 were used to dissect cell cycle progression. Fig. 1C showed that the expression of Erbin with a distinct molecular weight of 180 kDa varied during mitosis, rising in S phase and peaking in G2/M phase. Notably, the expression pattern was apparently coincident with that of cyclin B1, a known mitotic substrate of the anaphase promoting complex/cyclosome. To further validate the data, human cervical carcinoma cell line HeLa, human kidney cell line 293T, and immortalized human hepatic cell lines LO2 and HL-7702 were also treated with nocodazole. Similar data were obtained in all cell lines tested (Fig. 1D). These data indicate that Erbin is expressed in a cell cycle-dependent manner.

### The transcription of Erbin is controlled in a cell cycle-dependent manner

**Figure 2:**
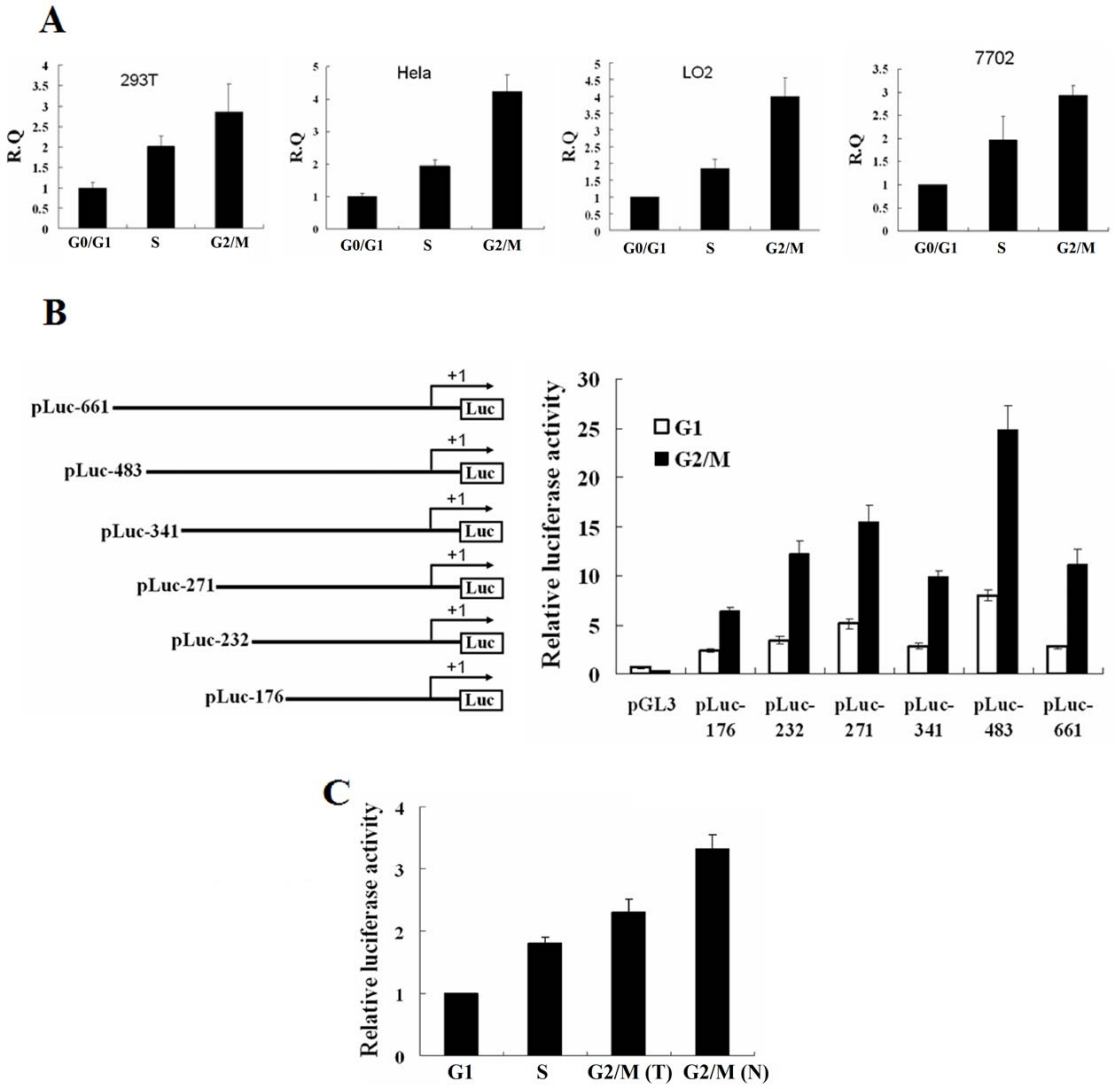
The transcription of *Erbin* is controlled in a cell cycle-dependent manner. A, The cells were arrested by a double-thymidine block or nocodazole treatment. Total RNA was extracted and *Erbin* mRNA expression analyzed by real-time RT-PCR. B, Schematic maps of the plasmids that carry the various *Erbin* promoter fragments driving the expression of luciferase (left). HeLa cells were transiently co-transfected with these plasmids and pRL-TK, then synchronized by a double-thymidine block and nocodazole treatment, respectively. The luciferase activities were assayed (right). C, HeLa cells were transiently transfected with pLuc-483, synchronized by double-thymidine block or nocodazole treatment, respectively, and then harvested at the different stages of the cell cycle. The luciferase assays were performed. (T), double-thymidine block; (N), nocodazole treatment.

We then determined whether the transcription of *Erbin* exhibits a similar change during cell cycle progression by examining the levels of the *Erbin* mRNA in the synchronized cell populations using real-time RT-PCR. The cells at different cell cycle stages were harvested, total RNA was extracted and *Erbin* mRNA expression analyzed. As shown in Fig. 2A, the Erbin mRNA was expressed at a low level in G0/G1 phase, but increased in S phase and reached a peak in G2/M phase in 293T, HeLa, LO2 and 7702 cells. To determine if the cell cycle-dependent transcription of the *Erbin* mRNA results from corresponding activation of the *Erbin* promoter and identify the critical region, which confers the cell cycle-dependent activation, in the *Erbin* promoter, we constructed the plasmid that carries the core region of the *Erbin* promoter (pLuc-661) and various deletions of the 5′-end of the *Erbin* promoter fragments driving the expression of luciferase (pLuc-483, pLuc-341, pLuc-271, pLuc-232 and pLuc-176) (Fig. 2B). HeLa cells were transiently co-transfected with these plasmids and pRL-TK and then synchronized by a double-thymidine block that arrests cells at G1/S boundary or by nocodazole treatment for G2/M phase arrest, respectively. The arrested cells were harvested and assayed for luciferase activity. In G1 phase cells, the luciferase activities were low, but the luciferase activities in the cells arrested in G2/M phase were dramatically enhanced about 2–4-fold. The promoter fragment containing a deletion up to nucleotide position −176 retained the basal activity of the *Erbin* promoter. The highest luciferase activity was observed in the cells transfected with pLuc-483, while a longer *Erbin* promoter fragment (pLuc-661) displayed less 50% of the promoter activity compared with the shorter form (pLuc-483) (Fig. 2B). To verify the cell cycle-specific transcriptional activation of *Erbin*, HeLa cells were transiently transfected with pLuc-483 and then synchronized by double-thymidine block or nocodazole, respectively. Following release at 0, 5 and 11 h, the cell lysates were prepared and luciferase assays performed. Fig. 2C showed that the *Erbin* promoter activity was low in G0/G1 phase (0 h post-release), but started to increase in S phase (5 h post-release) and achieved a maximum in G2/M phase (11 h post-release), consistent with the expression of Erbin mRNA. Similar results were also achieved in 293T cells (data not shown). The data suggest that the transcription of *Erbin* is regulated in a cell cycle-dependent manner.

### c-Myb binding site is critical for the cell cycle-dependent transcription of *Erbin*

**Figure 3:**
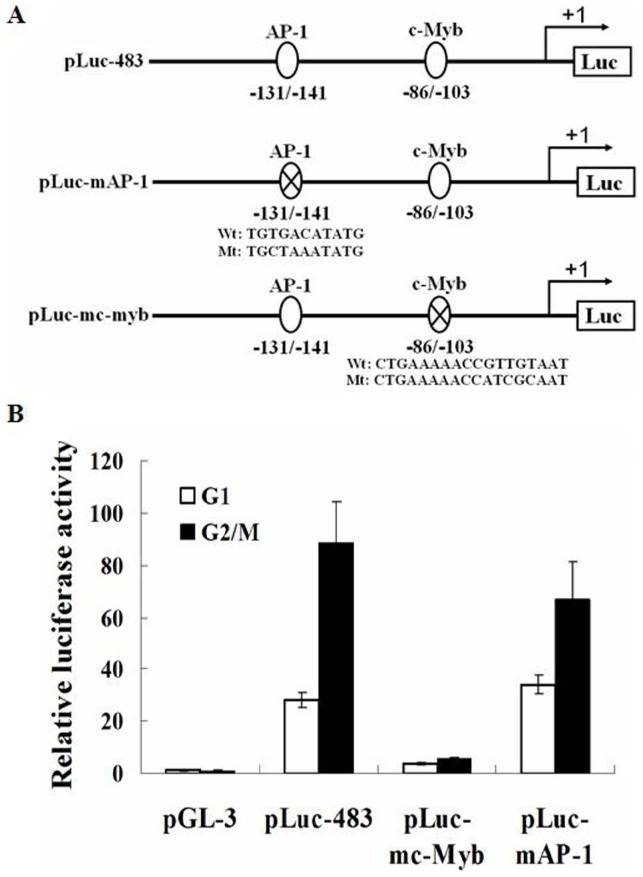
c-Myb controls cell cycle-dependent transcription of *Erbin*. A, Schematic maps of the plasmids that contain wild-type Erbin promoter fragment or fragments harboring substitutional mutations of the AP-1 and c-Myb consensus sequences. B, The luciferase activities in the transfected cells were determined. Wt, wild-type; Mt, mutant.

Sequence analysis revealed that the proximal region of the *Erbin* promoter includes a consensus AP-1 site at position −131/−141 and a c-Myb site at position −86/−103. The ability of AP-1 in regulating cell cycle progression is well known. A recent study demonstrated that c-Myb contributed to G2/M transition by direct regulation of cyclin B1 expression [22]. To clarify whether the regulatory elements in the *Erbin* promoter are responsible for the cell cycle-dependent transcription of *Erbin*, we introduced substitutional mutations in the AP-1 and c-Myb consensus sequences. TGACA was converted to CTAAA for AP-1 and GTTGT to ATCGC for c-Myb (Fig. 3A). The luciferase activities in the cells transfected with the construct containing the mutated AP-1 site was only slightly decreased in G2/M phase compared with the cells transfected with pLuc-483. Surprisingly, mutation of c-Myb binding site strikingly eliminated the luciferase activities, especially in G2/M phase (Fig. 3B), implicating that c-Myb may positively regulate the transcription of *Erbin*. In order to further characterize other enhancer elements, we also screened for evolutionarily conserved, potential transcription factor-binding sites in the *Erbin* promoter. Although a number of motifs, which are known to be specifically engaged in the periodic transcription of target genes, were identified, including Oct1 and Sp1, the involvement of these elements in the *Erbin* transcriptional activation was excluded by our experiments.

### c-Myb controls cell cycle-dependent transcriptional activation of Erbin

**Figure 4:**
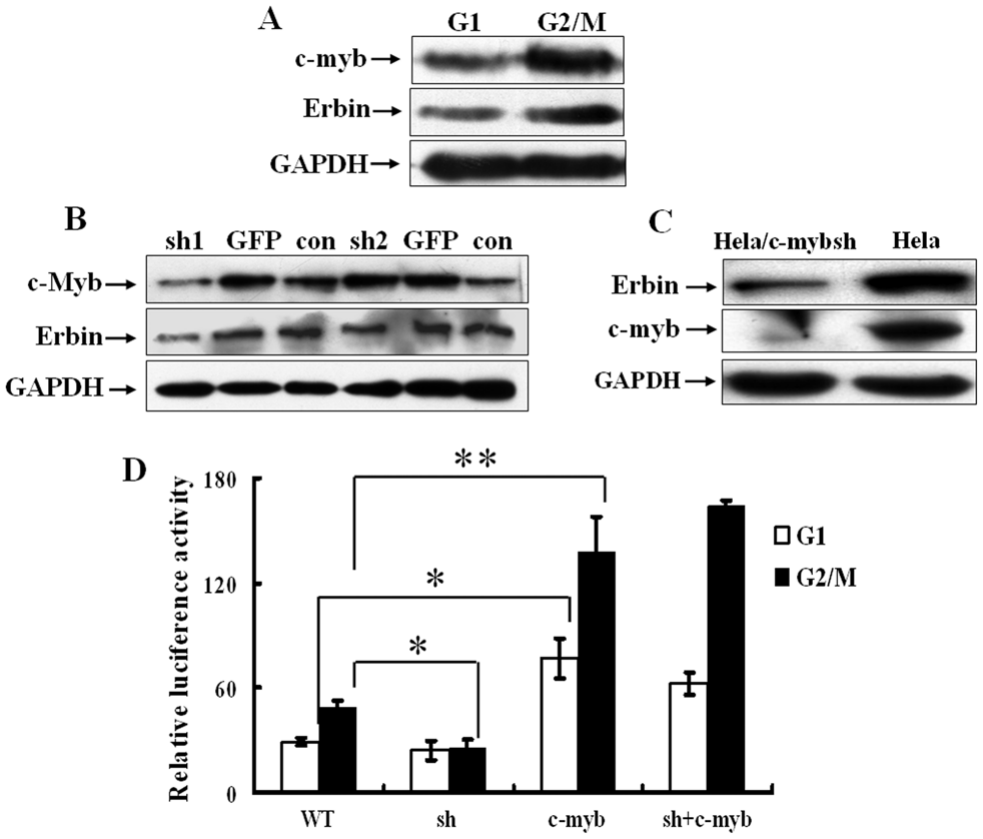
c-Myb level associates with Erbin expression. A, The expression of c-Myb and Erbin were analyzed by Western blot in the cells synchronized by double-thymidine block or nocodazole treatment, respectively. B, The expression of c-Myb and Erbin were examined by Western blot in the cells transiently transfected with the plasmids expressing c-Mybsh1, c-Mybsh2 and GFP shRNA. C, The expression of Erbin and c-Myb was detected by Western blot in the cells stably expressing c-Mybsh1. D, HeLa cells were transfected either with the plasmid expressing c-Mybsh1 or pHA-c-Myb or both. The transfected cells were arrested in G1 or G2/M phases, respectively, and the luciferase assays were performed.

c-Myb is a transcription factor that regulates cellular differentiation and proliferation [23]–[26]. A recent study demonstrated that c-Myb directly regulated cyclin B1 expression [22]. Above data showed that the expression pattern of Erbin during cell cycle progression was very similar to that of cyclin B1. It has been reported that c-Myb mRNA and protein have a very short half-life [22]. It implies that c-Myb may be distinctively expressed in G2/M phase if it exerts its regulatory function in this phase. We then detected c-Myb expression by Western blot. Fig. 4A showed that both c-Myb and Erbin levels were relatively low in G1 phase, but unequivocally and concordantly increased in G2/M phase.

To further confirm the correlation of c-Myb level with Erbin expression, we examined whether repression of c-Myb can decrease the Erbin expression. We designed two shRNAs specific for two different *c-Myb* target positions (c-Mybsh1 and c-Mybsh2) and cloned them into the plasmid pSuppressorNeo. Transfection with the c-Mybsh1 efficiently inhibited c-Myb expression, whereas c-Mybsh2 and control GFP-specific shRNA did not interfere with the c-Myb expression. Interestingly, silencing of c-Myb resulted in a significant reduction of the Erbin expression (Fig. 4B). The effect appeared to be specific, since the level of Erbin was not changed in the cells transfected with either GFP-specific shRNA or c-Mybsh2 (Fig. 4B). Thus, c-Mybsh1 was used in the following experiments. We established HeLa cells stably expressing c-Mybsh1. The data shown in Fig. 4C demonstrated that the Erbin protein was conspicuously declined concomitant with the knockdown of c-Myb expression. To determine if *c-Myb* silencing could influence the *Erbin* promoter activity, HeLa cells were transfected with the plasmid expressing c-Mybsh1 or pHA-c-Myb or co-transfected with both. The transfected cells were arrested in G1 and G2/M phases, respectively. As determined by luciferase assays, ectopic expression of c-Myb caused a marked increase of the *Erbin* promoter activities in the cells arrested in both G1 and G2/M phase. The shRNA-mediated knockdown of *c-Myb* did not significantly affect the *Erbin* promoter activity in G1 phase cells, but the luciferase activity in G2/M phase cells was drastically inhibited. Co-transfection with pHA-c-Myb and c-Mybsh1 effectively restored the *Erbin* promoter activity that was impaired by knockdown of *c-Myb* (Figure 4D). These data indicate that c-Myb controls cell cycle-dependent transcriptional activation of *Erbin*.

### c-Myb binds to the c-Myb consensus sequences in the *Erbin* promoter

**Figure 5:**
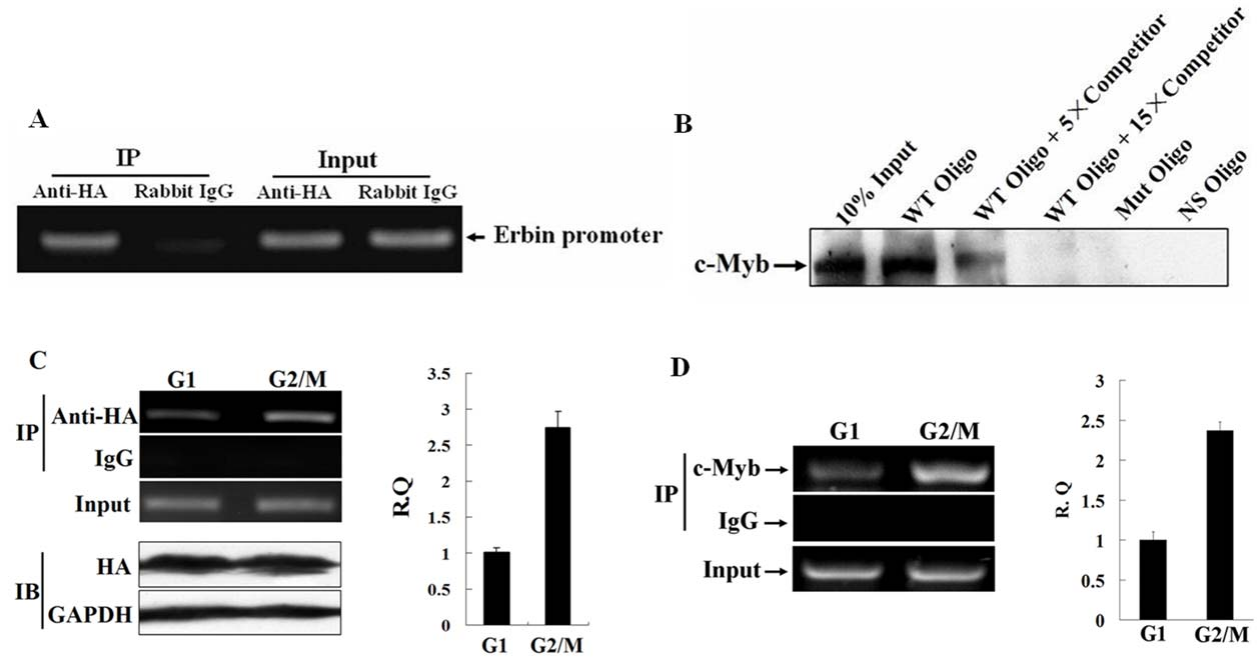
c-Myb binds to the c-Myb consensus sequences in the *Erbin* promoter. A, The association of c-Myb with the *Erbin* promoter in vivo was demonstrated by ChIP assays. B, The biotinylated double-stranded oligonucleotides harboring the consensus motif of c-Myb were incubated with 200 µg of the nuclear extracts and the binding of the nuclear proteins to the biotinylated oligonucleotides was analyzed in the presence or absence of five- and fifteen-fold amounts of double-stranded oligonucleotide competitors containing the c-Myb motif by DNA affinity precipitation assays. C and D, The binding activities of exogenous and endogenous c-Myb to c-Myb site in the *Erbin* promoter during cell cycle was analyzed by ChIP assays with the anti-HA (C) and anti-c-Myb antibodies (D) and real-time PCR.

We performed ChIP assays to test if c-Myb binds directly to the c-Myb binding site in the *Erbin* promoter *in vivo*. As shown in Fig. 5A, immunoprecipitation with the anti-HA antibody followed by PCR with the specific primers yielded a distinct band containing the c-Myb binding site. In contrast, immunoprecipitation with rabbit IgG resulted in the absence of this band, signifying the association of c-Myb with the *cis*-activating element in the promoter of *Erbin in vivo*.

To further testify the binding specificity of c-Myb to the c-Myb site in the *Erbin* promoter, we performed a DNA affinity precipitation assay. The biotinylated double-stranded oligonucleotides harboring the consensus motif of c-Myb were incubated with 200 µg of the nuclear extracts. The binding of the nuclear proteins to the biotinylated oligonucleotides was analyzed in the presence or absence of the double-stranded oligonucleotide competitors containing c-Myb motif. The association of c-Myb with the c-Myb consensus sequences could be reproducibly detected. The binding activity of c-Myb was remarkably inhibited by five-fold amount of the specific competitors. Competition with fifteen-fold amount of the competitors resulted in a complete loss of the biotinylated DNA/protein complexes (Fig. 5B). No binding of endogenous c-Myb to either the oligonucleotides containing a mutated c-Myb or nonspecific oligonucleotides was detectable. This experiment provides *in vitro* evidence that the binding of c-Myb to the c-Myb site in the *Erbin* promoter is specific. We then determined whether the binding affinity of c-Myb to the c-Myb site in the *Erbin* promoter varies during different phases of cell cycle. The data in Fig. 5C and 5D clearly confirmed that the binding affinity of either endogenous or exogenous c-Myb to the c-Myb site was significantly higher in G2/M phase than in G1 phase, indicating that both the binding affinity of c-Myb to the c-Myb motif and periodic variation of c-Myb level modulate cell cycle-dependent transcription of Erbin.

Taken together, our experiments define the functional role of c-Myb transcription factor in activating cell cycle-dependent transcription *of Erbin*.

### Erbin plays a critical role in cell cycle transition

**Figure 6:**
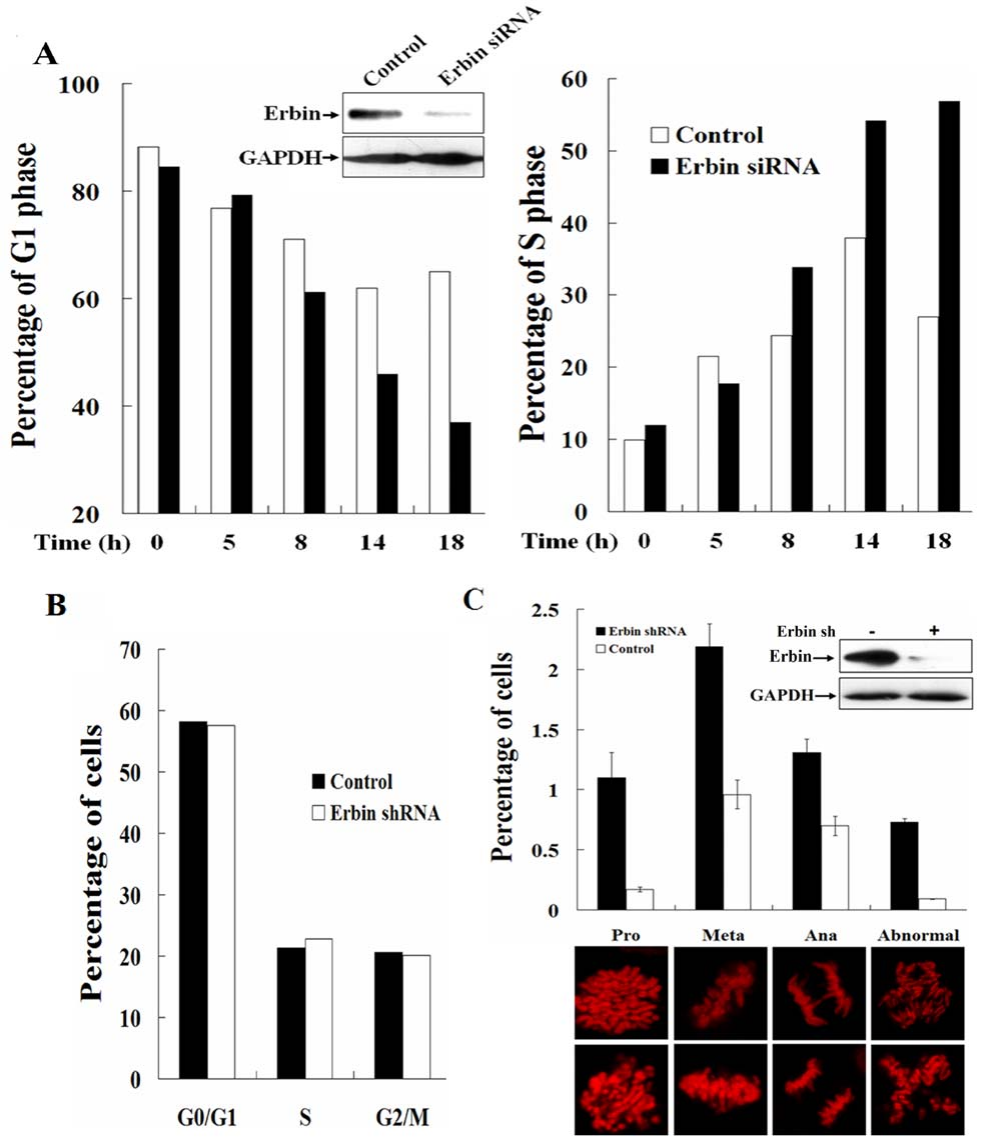
Erbin plays a critical role in cell cycle transition. A, LO2 cells were transiently transfected with Erbin-specific siRNA and synchronized by mimosine followed by release into nocodazole. Cell cycle progression was analyzed by flow cytometry. B, The cell cycle was analyzed by flow cytometry in both HeLa cells and HeLa cells stably transfected with Erbin specific shRNA. C, HeLa cells were fixed, stained by 5 µg/ml propidium iodide and photographed. Cells at mitosis were counted and divided by the total amount of observed cells.

Recent studies demonstrated that c-Myb contributes to G2/M cell cycle transition in human hematopoietic cells [22], [27]. It was also reported that the *c-Myb* antisense oligonucleotides inhibited T-cell proliferation in response to mitogens with a concomitant block to cell cycle progression in late G1 or early S phase and repressed the expression of cdc2 [28], which is required for entrance into mitotic phase, at the G1/S boundary [29]. c-Myb can directly regulates the expression of cyclin B1, which is important in regulating G1/S as well as G2/M cell cycle transition in hematopoietic cells [22]. Since the cell cycle-dependent expression of Erbin was regulated by c-Myb, it was reasonable to propose that Erbin is involved in cell cycle progression. In order to explore the physiological role of Erbin in cell cycle progression, we utilized LO2 cells that can be taken as human normal embryon hepatic cells. LO2 cells were transiently transfected with Erbin-specific siRNA to silence the expression of Erbin and then synchronized by mimosine, a potent reversible late G1 phase blocker, followed by release into the complete media containing nocodazole. Cell cycle progression was analyzed by flow cytometry. At 8 h after release from the block, the percentage of the cell population in G1 phase was significantly decreased and the S phase cells remarkably increased compared with untreated cells. The G1 phase cells were further decreased at 14 and 18 h, with concomitant increase of the S phase cells, whereas the change in the percentage of the G2/M phase cells was hardly seen at all time points (Fig. 6A). These data suggested that Erbin may regulate G1/S transition of the cell cycle.

To further test the role of Erbin in mitosis, we established Erbin knockdown HeLa cells, in which the function of Erbin was stably suppressed by the Erbin specific shRNA. The significant changes in cell cycle distribution were not detectable by flow cytometric analysis in these cells (Fig. 6B). However, when we focused on the cells in mitosis, we noticed that the majority of the mitotic cells were accumulated at prometaphase and metaphase by the morphological analysis (Fig. 6C). Although many chromosomes became bioriented and aligned at the metaphase plate, the formation of multipolar spindles was remarkably increased in the Erbin silenced cells. Despite these abnormalities, the cells with the suppressed functions of Erbin eventually entered anaphase with more than three chromosome masses. These data suggest that Erbin plays an important role in cell cycle progression.

## Discussion

The main findings of the present study are as follows: First, Erbin expression exhibits a feature of the cell cycle periodicity. The level of Erbin rises in S phase, peaks in G2/M phase and declines in G0/G1 phase. A significant increase in the *Erbin* promoter activity was observed in G2/M phase-synchronized cells, indicating that the variation in the *Erbin* mRNA levels throughout the cell cycle progression is based on the function of the *Erbin* promoter and that the expression of Erbin is controlled at the transcriptional level in a highly dynamic manner. The structure of the Erbin protein accords with the characteristics of short-lived proteins, many of which participate in the regulation of cell cycle progression [30]. The spatial and temporal regulation of the cell cycle depends on the elimination of these proteins by ubiquitin-proteasome pathway periodically. The level of the Erbin protein parallels that of cyclin B1, predicting its functional relevance to cell cycle regulation.

Second, c-Myb regulates cell cycle-dependent expression of Erbin. We noticed that Erbin expression varied directly with the c-Myb level, which is also controlled in a cell cycle-dependent manner. Sequence analyses revealed a c-Myb site in the core region of the *Erbin* promoter. ChIP assays, mutagenesis of the c-Myb consensus sequences and promoter pull-down assays validated the recruitment of c-Myb to the *cis*-acting element in the *Erbin* promoter. The interaction of c-Myb with the c-Myb site in the *Erbin* promoter was significantly enhanced in G2/M phase. Moreover, ectopic overexpression of c-Myb greatly enhanced the *Erbin* promoter activity and c-Myb silencing effectively repressed the Erbin protein level. It proves that c-Myb and the c-Myb motif in the core promoter of *Erbin* mediate the cell cycle-dependent transcription of *Erbin*. These results unravel a critical role of c-Myb in promoting *Erbin* transcription in G2/M phase.

Third, our study discloses an unappreciated function of Erbin in cell cycle progression. Our results suggest that Erbin may be involved in regulating G1/S transition. Inactivation of Erbin causes an acceleration of the G1/S transition and the formation of multipolar spindles. However, how Erbin acts in cell cycle progression is unclear. It has been shown that Ras/Raf/MEK/ERK signaling cascade plays a central role in promoting entry into S-phase by indirect regulation of the expression of cyclins and the activities of cyclin-dependent kinases during the cell cycle. ERK activation in G2-phase cells results in an increased number of cells containing chromosome aberrations. It has been demonstrated that Erbin inhibits the Ras-mediated activation of MAPK pathway. As a multiple-domain-containing scaffolding protein, it is conceivable that Erbin might exert a finely diversified regulation of Ras signaling by dynamically organizing multi-protein signaling complexes through its recognition domains during cell cycle progression. So far, the functional roles of Erbin are still largely unknown. Our findings provide a first hint to the novel functions of Erbin in mitosis.

The c-Myb protein is ubiquitously expressed in nearly all immature, proliferating hematopoietic precursors as well as several other cell types [31]–[34]. It has been reported that the c-Myb protein cooperates with a wide variety of other transcription factors in the transcriptional activation of target promoters, including p300/CBP, CCAAT binding protein family and Ets family proteins [35]–[40]. Enhanced transactivation of the Myb-responsive promoters was found when these cooperating factors are co-expressed with c-Myb. It has also been known that c-Myb function is also regulated by its association with other cell cycle proteins, such as c-Myc, and cyclin D1 [41]. The *Erbin* promoter may also under control of other transcription factors. The transcription factors that negatively regulate the transcription of Erbin is expected to exist. Notably, pLuc-483 exhibited a 2 fold increase in the luciferase activity over pLuc-661, indicating that potential *cis*-acting elements involved in negative regulation of the *Erbin* promoter is located within the −661 to −483 region. The related transcriptional factors have not been identified in the current study. Further studies will explicate more clearly the role of Erbin in cell cycle progression and molecular mechanisms of Erbin expression regulation.
